# The clinicopathological characteristics and survival outcomes of endometrial carcinoma coexisting with or arising in adenomyosis: A pilot study

**DOI:** 10.1038/s41598-020-63065-w

**Published:** 2020-04-06

**Authors:** Xiaopei Chao, Ming Wu, Shuiqing Ma, Xianjie Tan, Sen Zhong, Yalan Bi, Huanwen Wu, Jinghe Lang, Lei Li

**Affiliations:** 1 Department of Obstetrics and Gynecology, Peking Union Medical College Hospital, Beijing, 100730 China; 2 Department of Pathology, Peking Union Medical College Hospital, Beijing, 100730 China

**Keywords:** Endometrial cancer, Oncogenesis

## Abstract

Little is known about the epidemiological and clinicopathological characteristics of endometrial endometrioid carcinoma (EEC) coexisting with or arising in adenomyosis (EEC-A or EEC-AIA) due to their rarity. This study compared EEC-A and EEC-AIA with endometrial carcinoma without adenomyosis. Cases of endometrial cancer treated at the study center from June 1, 2010, to June 1, 2017, were reviewed. The epidemiological, clinicopathological characteristics and survival outcomes were compared among three groups of endometrioid subtypes: group A, stage IA endometrial carcinoma patients without coexisting adenomyosis; group B, patients with EEC-A; and group C, patients with EEC-AIA. Among the 2080 patients reviewed, groups A, B, and C included 1043, 230 and 28 patients, respectively. Patients in group A and group B had similar clinicopathological and survival outcomes. Patients in group C were significantly younger and had less gravidity and parity than patients in groups A and B. More tumors from group C were grade 1, and they had a smaller maximum diameter and less mismatch repair deficiency than those from groups A and B. After a median follow-up of 57.0 months, the 5-year disease-free survival (DFS) rates of groups A, B and C were 96%, 91% and 100% (*p* = 0.045), respectively; the 5-year overall survival (OS) rates were 98%, 93% and 100%, respectively (*p* = 0.001), in the Kaplan-Meier analysis. However, these difference disappeared in a subgroup of stage IA patients in univariate and multivariate analysis. Cox regression analysis in stage IA patients also revealed no significant differences in survival outcome across the three groups. In conclusion, EEC-AIA exhibited specific clinicopathological characteristics that were probably associated with favorable survival outcomes. The characteristics and survival outcomes of EEC-A were similar to those of EEC without adenomyosis in stage IA patients.

## Introduction

Endometrial cancer is estimated to be the fourth and the ninth most common cancer in terms of new cases and ranks sixth in cancer-related deaths in the United States^1^. In China, endometrial cancer (EC) is the ninth most common cancer and ranks tenth in cancer-related deaths, corresponding to 63,400 new cases and 21,800 deaths in 2015, respectively^2^. The overall survival of EC is usually better than that of other gynecologic malignancies, as most of these tumors are low-grade early-stage endometrial endometrioid adenocarcinoma (EECs)^3–5^. Adenomyosis (AM) is traditionally defined as the presence of ectopic endometrial glands and stroma within the myometrium and has been suggested to share some characteristics with malignant tumors, such as angiogenesis, abnormal tissue growth, and invasion^6–8^. AM has been observed in 10% to 70% of all hysterectomies based on its definition^9^.

AM is also considered a risk factor for endometrial and thyroid cancers^6^. In EC specimens, AM is sometimes found as a coexisting benign condition, with an incidence of 10% to 18%^10^. However, the role and involvement of AM in the pathogenesis and prognosis of EC is still unclear. In addition, the findings regarding the survival outcomes of patients with EEC without AM and those with EEC coexisting with AM (EEC-A) remain controversial. However, EEC arising in adenomyosis (EEC-AIA), i.e., malignant transformation from uterine adenomyosis, is extremely rare and estimated to occur in less than 1% of endometrial cancer cases^11^. Since Colman and Rosenthal firstly discovered EC-AIA in 1958^12^, only 46 and 78 cases have been reported by Machida *et al*.^11^ and Habiba *et al*.^13^ up to 2017, respectively, due to its strict definition. Available evidence for EC-AIA has been limited to case reports and exploratory analyses of the literature^14^. Due to the limited sample size and study design, no valid conclusions about the EC-AIA have been reached or generalized from these studies. Such limitations have hampered our understanding of the pathogenesis, evolution and management of EEC-A and EEC-AIA.

In this study, we reviewed the medical records of endometrial cancer patients treated at a tertiary teaching hospital from 2010 to 2017 to explore the clinicopathological features and survival outcomes of patients with EEC-AIA, EEC-A and EEC without adenomyosis.

## Materials and Methods

### Ethics approval

This is an observational retrospective cohort study. All patients provided consent before surgical treatment. The Institutional Review Board of Peking Union Medical College Hospital has approved this study (No. ZS-1428), and had also waived the need for informed consent to participate the study due to its retrospective nature. The registration number in *clinicaltrials.gov* is NCT03291275 (registered on September 25, 2017). All procedures performed in the study involving human participants were in accordance with the ethical standards of the institutional in the study center, and/or national research committee, and with the 1964 *Declaration of Helsinki* and its later amendments or comparable ethical standards.

### Study design

**Figure 1:**
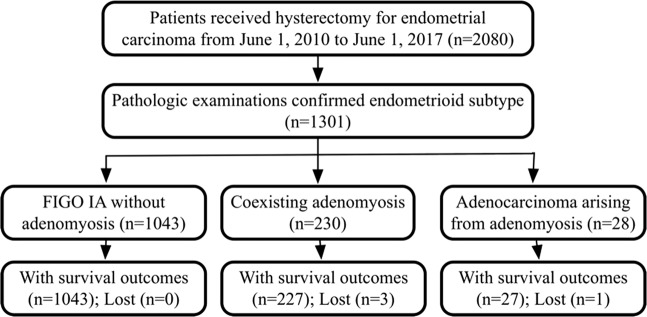
Flowchart of the study.

This retrospective cohort study was conducted in a tertiary teaching hospital. All eligible patients with EEC were reviewed and classified into three groups as follows: group A, patients diagnosed with International Federation of Gynecology and Obstetrics (FIGO) stage IA ECC without AM as a reference; group B, patients with EEC-A of all stages; and group C, patients with EEC-AIA of all stages (Fig. 1). The primary objectives were the differences in epidemiological and oncological characteristics among the three groups. The second objective consisted of survival outcomes, including disease-free survival (DFS) and overall survival (OS), and relevant risk factors.

### Study population

Pathological characterization was carried out in patients who underwent simple hysterectomy or comprehensive staging surgeries at the study center for primary endometrial cancer from June 1, 2010, to June 1, 2017. Two independent pathologists (HW and YB) reviewed all cases of EEC-A and EEC-AIA. Patients were excluded if their records indicated they had a non-endometrioid subtype or synchronous carcinomas of other sites. A cohort of stage IA EECs without AM of the same period was selected as a comparator (group A). Epidemiological, surgical and clinicopathological characteristics were collected via a specific database (Table 1 and Supplement 1). The metabolic diseases of the patients included diabetes, hypertension, hyperlipemia, overweight and obesity. The endometriosis found on the pathologic evaluation was classified as ovarian, peritoneal, or deeply infiltrating endometriosis (DIE).

**Table 1:** Epidemiological and clinicopathological characteristics of patients within three groups.

Parameter	Group A EEC without AM (n = 1043)	Group B EEC-A		Group C EEC-AIA		*P* value	
		All (n = 230)	Stage IA (n = 199)	All (n = 28)	Stage IA (n = 24)	Between three groups	Between stage IA patients of three groups
Age (year), mean ± SD	53.35 ± 9.97	54.50 ± 9.06	53.96 ± 8.99	48.39 ± 8.61	48.62 ± 9.19	**0.006**	**0.043**
Height (cm), mean ± SD	160.80 ± 5.89	161.11 ± 4.53	161.08 ± 4.58	159.86 ± 4.37	160.25 ± 4.55	0.488	0.714
Weight (kg), mean ± SD	67.44 ± 11.60	67.34 ± 10.93	67.36 ± 11.11	67.43 ± 14.16	67.21 ± 14.86	0.993	0.993
BMI (kg/m^2^), mean ± SD	26.13 ± 4.54	25.91 ± 4.01	25.92 ± 4.00	26.39 ± 5.66	26.17 ± 5.81	0.751	0.828
Menopause, n (%)	666 (63.85)	154 (66.96)	129 (64.80)	13 (46.43)	12 (50.00)	0.098	0.356
Metabolic disease, n (%)	395 (37.87)	86 (37.39)	71 (35.68)	14 (50.00)	12 (50.00)	0.416	0.386
Infertility, n (%)	20 (1.92)	4 (1.74)	4 (2.01)	2 (7.14)	1 (4.17)	0.142	0.735
Situation of fertility, n (%)							
Gravidity	2.42 ± 1.37	2.33 ± 1.26	2.34 ± 1.27	1.43 ± 1.17	1.50 ± 1.22	**0.001**	**0.004**
Parity	1.29 ± 0.90	1.30 ± 0.91	1.28 ± 0.93	0.71 ± 0.66	0.79 ± 0.66	**0.003**	**0.027**
Endometriosis*, n (%)	21 (2.01)	18 (7.83)	13 (6.53)	2 (7.14)	1 (4.17)	**<0.001**	**0.002**
Ovarian EM	10 (0.96)	13 (5.65)	8 (4.02)	2 (7.14)	1 (4.17)	**<0.001**	**0.003**
Peritoneum EM	11 (1.05)	12 (5.22)	10 (5.02)	2 (7.14)	1 (4.17)	**<0.001**	**<0.001**
DIE	9 (0.86)	10 (4.35)	9 (4.52)	0 (0)	0 (0)	**<0.001**	**<0.001**
Surgical routes, n (%)						**0.003**	**<0.001**
Laparoscopy	779 (74.69)	196 (85.22)	174 (87.44)	21 (75.00)	19 (79.17)		
Laparotomy	264 (25.31)	34 (14.78)	25 (12.56)	7 (25.00)	5 (2.51)		
Surgical procedures, n (%)						0.735	0.467
Simple hysterectomy	373 (35.76)	82 (35.65)	80 (40.20)	8 (28.57)	8 (33.33)		
Staging surgeries	670 (64.24)	148 (64.35)	119 (59.80)	20 (71.43)	16 (66.67)		
Ovarian preservation, n (%)	31 (2.97)	7 (3.04)	7 (35.18)	1 (3.57)	1 (4.17)	0.982	0.877
Differential of endometrioid EC, n (%)						**0.028**	**0.003**
Grade 1	706 (67.69)	176 (76.52)	159 (79.90)	24 (85.71)	21 (87.50)		
Grade 2	278 (26.65)	44 (19.13)	35 (17.59)	4 (14.29)	3 (12.50)		
Grade 3	59 (5.66)	10 (4.35)	5 (2.51)	0 (0)	0 (0.00)		
FIGO stages						**<0.001**	—
Stage I-II, n (%)	1043 (100)	220 (95.65)	199 (100.00)	25 (89.29)	24 (100.00)		
Stage III-IV, n (%)	0 (0)	10 (4.35%)	0 (0)	3 (10.71)	0 (0)		
Maximum diameter of the tumor (mm), mean ± SD	23.26 ± 18.02	20.53 ± 18.06	19.72 ± 18.79	18.29 ± 9.03	17.58 ± 8.96	**0.047**	**0.016**
Positive LVSI, n (%)	55 (5.27)	22 (9.57)	14 (7.04)	2 (7.14)	1 (4.17)	**0.046**	0.583
dMMR deficiency, n/n (%)	251/882 (28.46)	40/186 (21.5)	35/154(22.73)	4/28 (14.33)	3/20 (15.00)	**0.047**	**<0.001**
ER, n (%)	998 (95.68)	222 (96.52%)	189 (94.97)	27 (96.43)	24 (100.00)	0.837	0.518
PR, n (%)	1001 (95.97)	224 (97.39)	188 (94.47)	27 (96.43)	23 (95.83)	0.557	0.631
Postoperative adjuvant therapy, n (%)	84 (8.05)	34 (14.78)	14 (7.04)	4 (14.29)	1 (4.17)	**0.004**	0.707
Postoperative radiotherapy, n (%)	59 (5.66)	18 (7.83)	6 (3.02)	2 (7.14)	1 (4.17)	0.447	0.299
Postoperative chemotherapy, n (%)	36 (3.45)	16 (6.96)	5 (2.51)	3 (10.71)	0 (0)	**0.013**	0.525
Recurrence, n (%)	35 (3.36)	14 (6.17)	4 (2.00)	0 (0)	0 (0)	0.077	0.423
Recurrent sites, n (%)	33	13	4	0 (0)	0 (0)	0.184	0.954
Within the pelvic cavity	16 (1.53)	3 (1.32)	2 (50.00)	0 (0)	0 (0)		
Distant sites	17 (1.63)	10 (4.41)	2 (50.00)	0 (0)	0 (0)		
Mortality, n (%)	16 (1.53)	9 (3.96)	4 (2.00)	0 (0)	0 (0)	**0.041**	0.727
Mortality due to cancer, n (%)	11 (1.05)	6 (2.64)	3 (1.50)	0 (0)	0 (0)	0.135	0.743

Abbreviations: AM, adenomyosis; DIE, deep invasive endometriosis; dMMR, DNA mismatch repair; EEC, endometrial endometrioid carcinoma; EEC-A, endometrial endometrioid carcinoma coexisting with adenomyosis; EEC-AIA, endometrial endometrioid carcinoma arising in adenomyosis; ER, estrogen receptor; EM, endometriosis; LVSI, lymph-vascular space invasion; NA, not available; PALN, para-aortic lymph nodes; PR, progestrone receptor; SD, standard deviation.

*Some patients might have more than one subtype of endometriosis.

EEC-A in our study is defined as primary EEC coexisting with AM regardless of AM involvement. EEC-AIA was diagnosed according to the following diagnostic criteria utilized for the malignant transformation to ovarian cancer from endometriosis: (1) the carcinoma must not be situated in the endometrium or elsewhere in the pelvis; (2) the carcinoma must be determined to arise from the epithelium of adenomyosis and not to have invaded from another source; (3) endometrial stromal cells are observed to surround the aberrant glands to support a diagnosis of adenomyosis^12^; (4) there is evidence of transformation of the glandular structure from benign to malignant^15^; and (5) the carcinoma belongs to the endometrioid subtype. Based on the definition, in cases of EEC-A and EEC-AIA, the eutopic endometrium was reviewed and examined carefully to confirm whether it was involved or not.

### Interventions and follow-up

All patients consented to simple hysterectomy or comprehensive staging procedures by the judgement of clinicopathological factors, which included hysterectomy, bilateral salpingoophorectomy, and retroperitoneal lymphadenectomy. Postoperative adjuvant therapies followed relevant contemporary guidelines. All patients were closely followed until February 1, 2019, according to our customized protocol. In the follow-up protocol, the patients visited the outpatient clinics every 3 months for the first year after surgery, every 6 years for the next year, and yearly for the rest time. Recurrence was validated by physical examination, imaging and/or biopsy. The sites of recurrence were divided into categories within the pelvic cavity and distant sites. Mortality was confirmed by reviewing medical records and interviews by telephone and/or email. DFS was defined as the time interval between the date of hysterectomy and the date of the first recurrence of endometrial cancer or the last follow-up date without recurrence. OS was defined as the time interval between the date of hysterectomy and the date of death due to endometrial cancer or the last follow-up date if the patient was alive^11^.

### Statistics

Continuous variables exhibiting a normal distribution were compared using parametric methods. Categorical data and variables that did not exhibit a normal distribution were compared within three groups using nonparametric tests. Univariate analyses of survival were performed using the Kaplan-Meier method, and proportional hazards models were used to estimate the hazard ratios and 95% confidence intervals for whether AM was associated with DFS and OS. A multivariable analysis of DFS was performed using a Cox proportional hazard regression model with adjustment for statistically significant risk factors at baseline. All comparisons were performed with all patients and with only stage IA patients across all three groups. Unless otherwise stated, all analyses were performed with a two-tailed significance level of 0.05 and were conducted using SPSS 22.0 software (SPSS, Inc., Chicago, IL, USA).

Since there were only 28 patients of EC-AIA in our study, the statistic power (1-β value) of the analysis for survival outcomes is essential to determine the significance of this study. The statistic power was calculated with PASS 11.0 (NCSS, LLC. Kaysville, Utah, USA. www.ncss.com) using a non-inferiority testing model^16^ based on 5-year DFS or OS in stage IA patients. The 5-year DFS and OS of group A, i.e., EC without AM, were used as reference. The non-inferiority 5-year DFS and OS of stage IA group B and C patients were all defined as 95% and 96%, respectively. In this model, class I error probability (α value) was defined as 0.05. If the 1-β value> 0.90, the compare of 5-year DFS or OS was considered to be of enough statistic power.

### Ethics approval and registration

The Institutional Review Board of Peking Union Medical College Hospital approved this study (No. ZS-1428). The registration number is SOUM-1 (*clinicaltrials.gov*).

### Statement of submission

The paper is not under consideration by another journal, and the results presented in this work have not been presented or published previously.

### Key message

This large pilot cohort provided the comparison between endometrial endometrioid carcinomas coexisting with, arising in, and without adenomyosis. The detailed clinicopathological and survival outcomes provided the foundation of discussion on the relationship between endometrial cancer and adenomyosis.

## Results

### Demographic data of the study population

From June 1, 2010, to June 1, 2017, 2080 patients underwent hysterectomy or staging surgery for primary endometrial cancer. Five cases of EC-AIA and 12 cases of EC-A were excluded because they were non-endometrioid subtypes. The EEC-A and EEC-AIA groups (groups B and C) included 230 (11.06%) and 28 (1.35%) cases, respectively, and 1043 cases (50.14%) were confirmed to have stage IA ECC without AM (group A) (Fig. 1).

In the 230 patients in group B, there were 199 (86.5%), 19 (8.3%), 2 (0.9%), 2 (0.9%), 1 (0.4%), 6 (2.6%) and 1 (0.4%) cases of stage IA, IB, II, IIIA, IIIB, IIIC and IVB, respectively. Among the 28 patients in group C, there were 24 (85.7%), 1 (3.6%), 2 (7.1%) and 1 (3.6%) cases of stage IA, IB, IIIA and IIIC, respectively. Groups B and C had a similar stage distribution (*p* = 0.267), especially the proportions of stage IA and IB (*p* = 0.364).

### Comparison of epidemiological and clinicopathological characteristics

Table 1 shows the patient demographics and tumor characteristics of the patients in the three groups. Generally, compared with the patients in group A and group B, those in group C were younger and had less gravidity and parity, a higher proportion of their tumors were grade 1, and the tumors exhibited a smaller maximum tumor diameter and less mismatch repair (MMR) deficiency. These differences remained when the analysis was limited to all stage IA patients across the three groups. Only half (46.43%) of the patients in group C were postmenopausal, in contrast with two-thirds of the patients in groups A and B (63.85% and 66.96%), although this difference was not significant (*p* = 0.098).

Compared with the patients in group A, all group B patients (all stages or limited to stage IA patients) had similar epidemiological and clinicopathological characteristics, except that patients in group B had more endometriosis.

### Follow-up, overall survival and prognostic factors

A total of 1297 patients (99.69%) had definite survival outcomes over a median follow-up time of 57.0 months (range 3.8–105.4 months). Groups A, B and C had 35 (3.36%), 14 (6.17%) and 0 (0%) cases of recurrence, respectively, and 11 (1.05%), 6 (2.64%) and 0 (0%) cases of mortality due to cancer, respectively. No significant difference in terms of the site of recurrence was observed (*p* = 0.184). The median DFS interval was 57.70 (range 3.8 to 105.4), 50.30 (3.8 to 93.8) and 39.40 (24.1 to 93.2) months in groups A, B and C, respectively. The median OS interval was 58.40 (range 9.4 to 105.4), 50.90 (3.8 to 93.8) and 39.40 (24.1 to 93.2) months in groups A, B and C, respectively.

Of the patients in groups A, B and C, the 5-year DFS rates were 96%, 91% and 100%, respectively (*p* = 0.045); the 5-year OS rates were 98%, 93% and 100%, respectively (*p* = 0.001); and the 5-year cancer-specific OS rates were 98%, 95% and 100%, respectively (*p* = 0.030), in the Kaplan-Meier analysis. However, for stage IA patients in groups A, B and C, no significant differences were found in terms of the 5-year DFS rates (96%, 98% and 100%, *p* = 0.512), OS rates (98%, 98%, 100%, *p* = 0.422), or cancer-specific OS rates (99%, 98%, 100%, *p* = 0.575) due to the small sample size.

In stage IA patients, compares between group A and B, between group A and C, had statistic power (1-β) of 0.8249 and 1.000 for 5-year DFS, respectively; and had statistic power of 0.8698 and 1.000 for 5-year OS, respectively.

**Figure 2:**
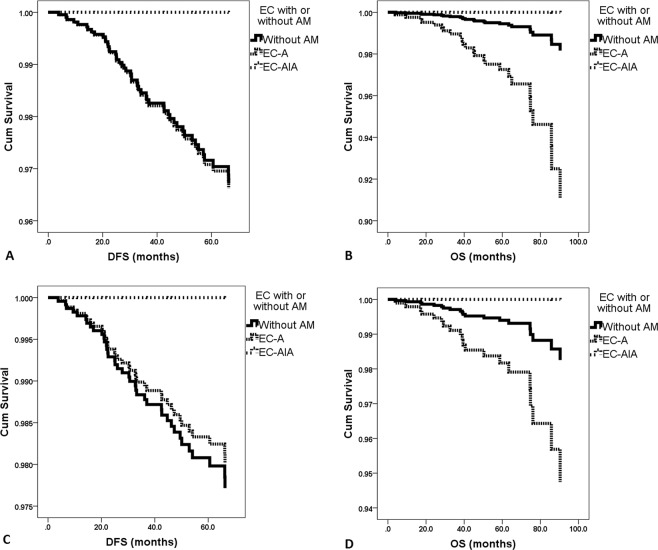
Survival outcomes of the enrolled patients according to Cox regression model. (**A**) The disease-free survival of the three groups. (**B**) The overall survival of the three groups. (**C**) The disease-free survival of stage IA patients from the three groups. (**D**) The overall survival of stage IA patients from the three groups. AM, adenomyosis. DFS, disease-free survival. EC, endometrial cancer. EC-A, endometrial cancer coexisting with adenomyosis. EC-AIA, endometrial carcinoma arising in adenomyosis. OS, overall survival.

We included age, co-existing endometriosis, surgical routes, differentiation, FIGO stages, the maximum diameter of the tumor, LVSI status and postoperative adjuvant therapy in the Cox regression mode (Table 2 and Fig. 2). As not all the patients had dMMR protein tested, it is not included in the model. In this model, for all patients, compared with group A patients (reference), EEC-AIA had similar DFS and OS; EEC-A patients had similar DFS but were associated with inferior OS (HR 5.033, 95% CI 1.803–14.048, p = 0.002). However, in this mode for stage IA patients, both EEC-AIA and EEC-A had similar DFS and OS compared with group A patients (Table 2).

**Table 2:** Multivariate analysis of clinicopathological characteristics of all cases for disease-free survival and overall survival.

	All patients				Stage IA patients			
	Disease-free survival		Overall survival		Disease-free survival		Overall survival	
	HR (95%CI)	*P* value	HR (95%CI)	*P* value	HR (95%CI)	*P* value	HR (95%CI)	*P* value
Groups		0.997		0.009		0.967		0.196
EC without AM	Reference	—	Reference	—	Reference	—	Reference	—
EC-A	1.029 (0.439–2.411)	0.947	5.033 (1.803–14.048)	0.002	0.869 (0.297–2.544)	0.797	3.066 (0.909–10.343)	0.071
EC-AIA	0.000 (0.000-N/A)	0.971	0.000 (0.000-N/A)	0.987	0.000 (0.000-N/A)	0.987	0.000 (0.000-N/A)	0.990
Age	1.021 (0.989–1.054)	0.208	1.003 (0.957–1.051)	0.902	1.016 (0.982–1.051)	0.360	1.001 (0.954–1.051)	0.967
Gravidity	1.016 (0.681–1.516)	0.938	0.816 (0.478–1.393)	0.456	0.941 (0.620–1.431)	0.777	0.798 (0.451–1.414)	0.440
Parity	0.992 (0.759–1.295)	0.951	1.277 (0.899–1.812)	0.172	1.082 (0.815–1.437)	0.587	1.288 (0.886–1.870)	0.184
Co-existing endometriosis								
No	Reference	—	Reference	—	Reference	—	Reference	—
Yes	0.964 (0.217–4.289)	0.962	0.000 (0.000-N/A)	0.978	0.000 (0.000-N/A)	0.980	0.000 (0.000-N/A)	0.984
Surgical route								
Laparoscopy	Reference	—	Reference	—	Reference	—	Reference	—
Laparotomy	0.684 (0.360–1.300)	0.247	0.251 (0.100–0.632)	0.003	0.443 (0.222–0.884)	0.021	0.214 (0.079–0.577)	0.002
Differential of endometrioid EC		0.031		0.438		0.046		0.805
Grade 1	Reference	—	Reference	—	Reference	—	Reference	—
Grade 2	1.675 (0.829–3.386)	0.151	0.899 (0.290–2.785)	0.853	2.164 (1.018–4.601)	0.045	1.211 (0.398–3.682)	0.736
Grade 3	3.173 (1.342–7.501)	0.009	2.048 (0.560–7.485)	0.279	3.083 (1.154–8.235)	0.025	1.694 (0.345–8.312)	0.516
FIGO stages								
Stage I-II, n (%)	Reference	—	Reference	—	—	—	—	—
Stage III-IV, n (%)	6.115 (1.897–19.712)	0.002	0.831 (0.138–5.000)	0.840	—	—	—	—
Maximum diameter of the tumor	1.009 (0.995–1.023)	0.206	1.009 (0.992–1.026)	0.286	1.003 (0.987–1.019)	0.696	1.009 (0.992–1.027)	0.312
LVSI								
Negative	Reference	—	Reference	—	Reference	—	Reference	—
Positive	1.753 (0.778–3.950)	0.176	1.450 (0.366–5.750)	0.597	2.235 (0.874–5.717)	0.093	2.030 (0.408–10.105)	0.387
Postoperative adjuvant therapy								
No	Reference	—	Reference	—	Reference	—	Reference	—
Yes	3.547 (1.640–7.672)	0.001	2.749 (0.820–9.214)	0.101	3.075 (1.367–6.919)	0.007	1.707 (0.422–6.908)	0.453

**Abbreviations:** AM, adenomyosis; EC, endometrial cancer; EEC-AIA, endometrial endometrioid carcinoma arising in adenomyosis; FIGO, Federation International of Gynecology and Obstetrics; HR, hazard ratios; 95% CI, 95% confidence interval; LVSI, lymph-vascular space invasion; N/A, not available.

In the Cox model, compared with EEC-A patients (reference), EEC-AIA had similar DFS and OS in patients of all stages (both HRs were 0.000, 95% CI 0.000-not available, p = 0.971 and 0.985, respectively), and in stage IA patients (both HRs were 0.000, 95% CI 0.000-not available, p = 0.989 and 0.987, respectively).

## Discussion

The potential relevance of endometrial carcinoma and AM is an attractive research topic, as it could reveal not only the relationship between AM and eutopic endometrium but also the pathogenesis of the malignant transformation of ectopic endometrium. To the best of our knowledge, this is the first report of a large pilot cohort of EEC-AIA and EEC-A. In our study, a 1.35% prevalence of EEC-AIA was documented in this large cohort after extensive pathological review, which parallels an approximately 0.93% prevalence of atypical glandular hyperplasia transformation of adenomyosis reported in a study conducted at another Chinese center^17^. In addition, according to the report of Kucera *et al*.^18^ malignant changes in adenomyosis were present in 6.8% (6/88) of patients with endometrial cancer, with different stages of hyperplastic changes observed. Little is known about the pathogenesis of EEC-AIA. The malignant transformation of adenomyosis is thought to be due to the transition of the endometrial epithelium into monolayer tumor cells^19^, which can produce many histological types, including EEC, papillary serous carcinoma^20^, serous carcinoma^21,22^, primary uterine müllerian mucinous borderline tumor (MMBT)^23^, and clear cell carcinoma^24^. The predominant histological types are EEC and clear cell carcinoma^7,25,26^. It has been suggested that cancer initially occurring within the myometrial layer can easily reach the myometrial stroma due to the lack of an anatomical barrier in the basal layer of endometrium^27^. However, these hypotheses have no valid supporting evidence, and a meta-analysis found that adenomyosis may not contribute to the development of myometrial invasion in endometrial adenocarcinoma^28^. Habiba *et al*.^13^ reported 78 cases of EC-AIA collected in 68 articles between 1897 and 2017. It is difficult to determine the exact number of cases reported in literature as diagnosis of many of the cases has been disputed^13^. Machida *et al*.^11^ used 46 cases for survival analysis. The low incidence rates and difficulty in preoperative imaging evaluation of EEC-AIA or EEC-A have hindered the development of a prospective trial^29^.

In our large cohort study, both EEC-A patients and stage IA EEC patients had good prognoses, but EEC-AIA patients had the best survival outcomes. However, in the Cox regression model, the difference of survival outcomes between EEC-A and EEC-AIA patients had no statistic significances, probably due to the limited sample size in EEC-AIA. The survival outcome of EC-AIA in our study is different with previous report. In a pooled analysis by Machida *et al*.^11^, 46 and 350 cases of EC-AIA and EC-A were compared, and they recovered that EC-AIA had distinct tumor characteristics and a poorer survival outcome compared to EC-A. The authors asserted that EC-A and EC-AIA were unique entities^11^. But their study design had several limitations. First, EC-AIA and EC-A were collected by systematic literature search and a historical cohort, respectively. The inconsistence of study subjectives probably had essential impact on their conclusions. Second, in the study of Machida *et al*.^11^, type II EC had 4 (8.7%) and 33 (9.4%) cases in EC-AIA and EC-A groups, respectively. Various stages of EC were also illustrated. These important bias would interfere with the analysis for survival outcomes. Hence, more evidences are needed to clarify whether EEC-A and EEC-AIA were two distinct pathological entities. Indeed, criteria for identifying and separate the EC-A and EC-AIA have been laid down and should be strictly followed. These criteria have been debated for more than half a century^30^. In spite of these clear and valid criteria listed in the text, attribution of cases remains problematic. The different biological behavior of EEC-AIA and EEC-A may have various underlying molecular mechanisms. Despite a few genetic studies on the pathogenesis of adenomyosis^28,31^, the transformation process requires greater analysis of cancer tissues, adenomyosis specimens (tissues adjacent to cancer), and normal endometrium. A thorough bioinformatics analysis would probably reveal the pathogenesis of AM transformation. A multiomics study of AIA is ongoing at our center (NCT04010487). However, due to the low incidence and strict definition of EEC-AIA, fresh specimens rather than paraffin sections are very difficult to harvest during surgery.

In our report, EEC-AIA exhibited specific characteristics that were nonetheless different from those reported previously. In our patients, EEC-AIA was associated with significantly younger onset ages and better survival than other subtypes, as no recurrence or death occurred in EEC-AIA patients. Although EEC-AIA had similar expression of progesterone and estrogen receptors in our study, in two exploratory analyses utilizing the EEC-AIA from data in the literature and non-EEC-AIA cases from a historical cohort at the studied centers^11,14,19^, EEC-AIA patients were found to be significantly older and less likely to express the estrogen receptor. The reasons behind the differences in the clinicopathological and survival outcomes of EEC-AIA require further clarification. Aromatase activity in adenomyosis lesions is higher than that in the normal muscle layer and normal endometrium^32^; thus, peri-menopausal women with adenomyosis may have relatively high estrogen states. As our study revealed that the mean age of EC-AIA patients was 48.39 years, it seems possible that high estrogen states in the peri-menopausal period induce malignant transformation. Although tamoxifen is an anti-estrogen drug, it sometimes causes high estrogen states, and EC-AIA has been reported in several patients during the treatment of oral tamoxifen^20,33^. However, in our study, the history of utilizing tamoxifen was not clear. The ability of estrogen to pathogenically stimulate endometrial tissue is well established, and estrogenic effects on the endometrium can lead to adenomatous and atypical hyperplasia; similar changes have been found in adenomyotic glands. Even a short duration of estrogen-only hormonal replacement therapy can induce malignant transformation within 2 years^34^. In our study, the younger average age of diagnosis and peri-menopausal status in EEC-AIA compared with EEC without adenomyosis is consistent with the above hypothesis. Previous reports have considered elderly age or postmenopausal status as epidemiological characteristics of EEC-AID^19^, likely due to the limited sample sizes and differences in the study designs.

In particular, we found that patients with EEC-AIA had significantly lower MMR deficiency with other EECs. MMR proteins are responsible for excising DNA mismatches introduced by DNA polymerase, and deleterious mutations of MMR genes contribute to Lynch syndrome, the most common hereditary syndrome pertinent to EC^35^. during cell division MMR protein expression has never been revealed in previously reported EEC-AIA patients, and its significance remains unclear. MMR deficiency was reported to be associated with improved outcomes in patients with nonmetastatic endometrial cancer^36^. However, other authors reported contrasting findings^37,38^, and the prognosis of Lynch syndrome-associated endometrial cancer did not appear to be different from that of sporadic tumors^39^. A detailed molecular analysis including Lynch syndrome-associated targeted gene sequencing is essential to explain these differences. The ER expression in EEC-AIA has been described in a few reports, which documented surprisingly low ER expression (14.3% compared with 84.6% in other endometrial cancer in Matsuo *et al*.^14^ and versus 93.4% in Machida *et al*.^11^). The reliability and repeatability of their reports is questionable because the data on EEC-AIA were collected by a literature review. However, as our study spanned eight years, the reliability of immunochemical evaluation was not completely agreed upon or fully integrated.

In our study, coexisting AM had no impact on the oncological characteristics or DFS of patients, who shared similar epidemiological factors with patients with EEC without AM. Although EEC-A had inferior OS, but the difference disappeared in stage IA patients. In 2216 patients awaiting placement of the levonorgestrel-releasing intrauterine system at our center, endometrial biopsy revealed 18 cases (0.81%) of cancer or intraepithelial neoplasia^40^. The presence of AM did not seem to have a significant influence on the prognosis of EEC^41^. In a small cohort of 82 cases, Hanley *et al*.^42^ even found low-stage EEC involvement of the deeply located AM does not affect patient prognosis. However, others revealed that the presence of AM in EC is associated with improved survival in endometrial cancer^43–45^. The inflammatory and tissue responses arising around the foci of adenomyosis generate a preventive mechanism against the invasion of adenocarcinomas coexisting with adenomyosis^46^. This response is likely the primary mechanism responsible for the good clinical course of these tumors^46^. In contrast, Taneichi *et al*.^41^ documented a high incidence of deep muscle invasion among cases of stage I EEC with AM. Some authors even suggested that the intraoperative evaluation of the presence of AM in patients with EEC may aid surgeons in estimating oncological risk and in selecting the most appropriate surgical treatment^47^.

The main strength of this study was its large sample size. However, one of the limitations of this study was the sample size of the EEC-AIA group. As there were only 28 and 24 cases in all and stage IA EEC-AIA patients, the limited sample size would probably cause bias interfering with the significances of statistics. Hence we proposed an non-inferiority analysis for 5-year DFS and OS, and defined strict cut-off values of statistic power (0.900). As a result, we achieve enough statistic power, which guaranteed the reliability of survival analysis. In addition, no targeted gene sequencing was performed to illustrate the prevalence of Lynch syndrome despite the various expression patterns of dMMR genes. As EEC patients have a favorable prognosis, long-term follow-up is needed to reveal the differences in survival outcomes. Cases of non-endometrioid subtypes complicated with AM warrant further exploration.

## Conclusions

In contrast with previous reports, in this large pilot study, EEC-AIA patients exhibited specific clinicopathological characteristics that were probably associated with improved survival outcomes. However, no significant differences were found between patients with EEC-A and those with ECC without adenomyosis in terms of epidemiological, pathological characteristics or prognosis, except that patients with ECC without adenomyosis had a higher proportion of coexisting endometriosis.

## Supplementary information

Supplementary information

## Data Availability

All datasets generated for this study are included in the article/Supplementary Material.
